# Technology and psychotherapeutic interventions: Bibliometric analysis of the past four decades

**DOI:** 10.1016/j.invent.2021.100425

**Published:** 2021-07-09

**Authors:** Arya Zale, Meagan Lasecke, Katerina Baeza-Hernandez, Alanna Testerman, Shirin Aghakhani, Ricardo F. Muñoz, Eduardo L. Bunge

**Affiliations:** a Department of Psychology, Palo Alto University, United States; b Institute for International Internet Interventions for Health, Palo Alto University, United States

**Keywords:** Bibliometrics, Publishing trends, Psychotherapy, Technology, Terminology

## Abstract

**Background:**

Rapid growth of the integration of technology and psychotherapeutic interventions has been noted, but no clear quantification of this growth has been done.

**Aims:**

This bibliometric analysis seeks to quantify the growth, trends, and applications of technology in psychotherapeutic interventions over the last 40 years.

**Methods:**

Searches were conducted in the Web of Science (WOS) database for all existing technology-psychotherapy-related publications from 1981 to October 2020. Search terms were refined using a systematic screening strategy, based upon Cochrane protocol, generating 52 technology terms. Analyses across 40 years and by decade from 1981 to 2020 were conducted.

**Results:**

A total of 13,934 peer-reviewed articles were identified. Yearly publication rate has increased from one in 1981 to 1902 by October 2020. The growth rate of publications across decades consistently tripled in size (762.50% from the 1980s to 1990s, 539.71% from the 1990s to 2000s, and 337.24% from the 2000s to 2010s). The author, country, journal, and institution with the most publications were Andersson, G., USA, Journal of Medical Internet Research, and Karolinska Institute, respectively. The most frequent technology search term across all four decades was “internet*.” The trends in percentages of peer-reviewed publications within each decade showed: 1) a declining trend for the term “computer,” 2) an upward trend for the combined terms, “internet,” “online,” and “web,” 3) and a steady but smaller proportion of publications for other terms (“cell phone,” “phone/telephone,” “technology,” “video,” “virtual reality or VR,” “apps,” “digital,” “machine learning,” “electronic,” “robo,” and “telehealth”).

**Discussion:**

The rapid growth and trends identified in technology and psychotherapy publications can inform related policies addressing the role of technology in mental health. Moreover, pattern analyses may provide direction for a standard nomenclature to address terminology usage inconsistencies across the field.

## Highlights

• This study quantifies growth of technology use in psychotherapeutic interventions.
• Publications on psychotherapy and technology tripled in size across each decade.
• Publications utilizing the terms “internet/web/online” are continually growing.
• The use of the term “computer” is decreasing.
• Other technologies appear at lower frequencies but remain relatively stable.

## 1 Introduction

Examining variations in publications across time can reveal trends related to the evolution of a field and can contribute to the development of hypotheses for future directions (Soares et al., 2020). A bibliometric analysis quantifies and synthesizes academic publications in a particular domain, contributing to scientific understanding in and across fields (Guo et al., 2020; OECD, 2013). A bibliometric analysis on publication trends in psychotherapy has identified the dominance of some psychotherapy brands across five decades and how societal changes, such as the emergence of Information and Communication Technologies (ICTs), may influence the delivery models of psychotherapy (Soares et al., 2020). While the integration of technology and psychotherapy may seem evident, to date, no bibliometric analysis has been conducted. A comprehensive understanding of the phenomenological relationship between technology and psychotherapy may contribute to the direction of the field and inform related policies addressing the role of technology in mental health.

In recent decades, the integration of technology and psychotherapy has been influenced by several factors: 1) the rapid growth of ICTs; 2) the societal adoption of these technologies; 3) the interest of psychotherapy researchers; 4) the support from funding agencies;5) mental health policies; and 6) historical events. These factors are described below.

First, the rapid growth of ICTs has been accompanied by the massive adoption of such technologies. For example, the first personal computers became available in the 1970s, with the first Apple computer in 1976 and the IBM PC in 1981 (Huddleston, 2019). By 2008, one billion personal computers were sold globally (Computers Reach One Billion Mark, 2002). By 2015, it is estimated that there were 1.5 billion personal computers in use (Statista Research Department, 2016). Such advances elicit a clear interest from the industry in applying technological advances to mental health. For example, by 2019 there were more than 315,000 apps related to mobile health (Gratzer and Goldbloom, 2019).

Second, the World Wide Web has facilitated access to increasingly large numbers (and proportions) of the world's population: 16 million (0.4%) in 1995, 36 million (5.8%) in 2000, 1.9 billion (29%) in 2010, and 4.8 billion (62%) in 2020 (Internet Growth Statistics, 2020). The first internet-delivered psychological treatment was conducted in 1996, only five years after the World Wide Web became publicly available (Smith and Bloom, 2019). Additionally, in 1996, Colón (1996) was the first to introduce the term “therapy online” to describe a therapeutic online chat group.

Third, the interest of psychotherapy researchers sparked the creation of two influential societies to promote scientific study of technology applied to evidenced-based interventions for behavioral and mental health; the International Society for Research on Internet Interventions (ISRII) formed in 2004 (Who We Are, 2015), and the European Society for Research on Internet Interventions (ESRII) developed in 2012 (Vlaescu, 2012). Furthermore, a whole body of journals dedicated to integrating technology and psychotherapy was developed, including the *Journal of Medical Internet Research* (JMIR) in 1990, and *Internet Interventions* in 2014 (Andersson et al., 2013).

Fourth, funding agencies have started to bolster research efforts in the application of technology to mental health. The National Institute of Mental Health (NIMH) has prioritized interest in obtaining grant applications utilizing technology to progress the assessment, identification, prevention, treatment, and provision of psychological services (NIMH, 2018). Increased access to psychotherapy has additionally been funded and prioritized by the National Health Service in Britain (Marks, 2017).

Fifth, government agencies such as the National Institute of Health (NIH) in the USA and various others globally have established mental health policies recommending dissemination of digital interventions (Ritterband et al., 2006). For example, beginning in 2006, the UK's National Institute for Health and Clinical Excellence (NICE) recommended “Beating the Blues,” a computerized Cognitive Behavioral Therapy (cCBT) treatment program for people with mild or moderate depression (*Online CBT Course | Therapy Online*, 2020; Ritterband et al., 2006).

Sixth, historical events demonstrate a substantial capacity to influence the rapid adoption of technology applications in mental health. For example, due to the COVID-19 pandemic, there was an over 12-fold upsurge from 7.07% to 85.53% of psychologists in the United States administering a portion of clinical work via telehealth (Pierce et al., 2020).

Amidst the growing integration of technology within mental health, researchers utilized a variety of terms to name their interventions. The abundance of new nomenclature led to a chaotic use of terminology in psychological research literature (Andersson et al., 2019; Barak, 2013; Smoktunowicz et al., 2020). There is no clear consensus on how to group and distinguish interventions integrating psychotherapy and technology (Smoktunowicz et al., 2020). Heterogeneity in platforms, technical features, human interaction, and other salient aspects of technological interventions contributes to overlapping and interchangeable terminology (Smoktunowicz et al., 2020). Moreover, the growing vocabulary generated an extensive aggregation of labels to organize the technological interventions, such as behavioral intervention technologies (BITS) (Mohr et al., 2015), internet interventions (Andersson et al., 2013), e-mental health (Riper et al., 2010), digital health interventions (DHIs) (Hollis et al., 2017), teletherapy (Schoenberg et al., 2008) and, web-based interventions (O'Leary et al., 2019). Inconsistency and unclear associations between terms and their corresponding applications creates obstacles when conducting systematic reviews, and impacts communication between researchers, practitioners and clients, policy-makers, the general population, and the media (Andersson et al., 2019; Smoktunowicz et al., 2020).

The current study was designed to gain a comprehensive overview of the number of publications on the intersection of technology and psychotherapy to identify: 1) the evolution of the amount of publications over the last 40 years; 2) the top fifteen most published authors, journals, countries, and institutions, addressing psychotherapy and technology over the last 40 years; 3) the most published technology terms over the last 40 years; and 4) the top fifteen most published technology terms throughout the periods of 1981-1990, 1991-2000, 2001-2010, and 2011-2020. This bibliometric study addressing both technology and psychotherapy has the potential to inform related policies addressing the role of technology in mental health and may clarify the extent of the problem and support the need for improved, efficient nomenclature.

### 1.1 A note on terminology

We are using the term “psychotherapy” in a very broad sense, that is, in the sense of interventions intended to have therapeutic effects. In its traditional sense, “psychotherapy” involves a therapeutic contract between a mental health provider (the therapist) and a patient or client. There are certain understandings between the two, including working mutually to alleviate a mental, emotional, or behavioral condition that is interfering with the client's life. Technically, then, digital interventions that do not involve a therapeutic contract are not “psychotherapy.” We have chosen to include digital interventions that have a clear therapeutic intent in our review, regardless of if the therapeutic intervention is implemented by a live therapist, a programmable digital tool (e.g., an app or a website), or a combination of live therapist and digital tools.

## 2 Methods

### 2.1 Search and screening strategy

To ensure consistency in methodology, three researchers conducted a systematic process, outlined by Cochrane, for running the searches (Lefebvre et al., 2008). All searches were conducted between July 2020 and October 2020 using the Web of Science (WOS) platform and the following citation indices were selected: Science Citation Index Expanded (SCIE) and Social Sciences Citation Index (SSCI). The non-aggregated SCIE and SSCI indices were chosen above comprehensive aggregated collections (such as “All Databases”) in order to optimize recall, precision, and reproducibility (Gusenbauer and Haddaway, 2020). The searches were conducted for the period from 1981 to October 2020. The following inclusion criteria guided keyword refinement: 1) the study involves the use of technology; 2) the study targets mental health or well-being, or pain or comorbid mental/medical condition; 3) the technology is applied to or utilized by human beings; and 4) the technology is used to inform or conduct the intervention or treatment for a mental health condition or the technology is used as the means to assess a mental health condition.

For the selection of psychotherapy and technology search terms, a list of search terms from systematic reviews and meta-analyses in the field of psychotherapy and technology was created (Appendix A). Six psychotherapy terms were selected (“clinical*” OR “counsel*” OR “intervention*” OR “psychotherap*” OR “therap*” OR “treatment*”). The mental health disorder search terms were chosen based on global prevalence rates of mental health disorders (*GBD Compare | IHME* Viz *Hub*, 2020; James et al., 2018) and included a total of 16 search terms (e.g., “anxiety” OR “depres*” OR “dysthymia” OR “substance use” OR “drug use disorder*” OR “intellectual disability” OR “attention deficit and hyperactivity disorder*” OR “conduct disorder*” OR “bipolar” OR “alzheimer*” OR “dementia*” OR “autism” OR “schizophrenia” OR “eating disorder*” OR “anorexia” OR “bulimia”).

For the technology terms, an initial pool of 430 terms was created based on the search terms of the systematic reviews. Some terms were merged (e.g., “virtual reality” OR “VR”), non-relevant or broad terms were removed (e.g., obesity, diet, BMI), and a few additional terms were added (e.g., “advanced technolog*”, “machine learning”, “tele health OR tele-health OR telehealth”, “telepsych*”, “telepthera*”). Additional descriptors were added to the search terms in order to aid in specificity [e.g., “electronic*” (NOT “health record*” or “medical record*” or “electronic record*”)]. After removing duplicates and merging similar terms, a list of 117 terms remained. From this list, only 52 search terms yielded ten or more articles and were retained for the current bibliometric analysis (see Appendix B for a complete list of Boolean search terms).

### 2.2 Analysis

The analysis for this bibliometric study used HistCite software and Excel frequency calculations. The overall amount of publications on psychotherapy and technology per year and the growth rate were analyzed for all 52 technology terms over the last 40 years. The growth of publications within each decade and across decades was calculated. After that, the total number and percentages of articles published across all four decades combined and per decade were analyzed. Next, the total and percentages of fifteen authors, journals, countries, and institutions with the most publications across all four decades were reviewed. The total and percentages of the fifteen technology terms with the largest number of publications were reported across all four decades combined and per decade. Finally, trends in the percentage of peer-reviewed publications within each decade across the last three decades were analyzed for the top fifteen technology terms overall.

## 3 Results

### 3.1 Descriptives

Using the psychotherapy, technology, and mental health disorder search term lists for the 52 technology terms, a total of 13,934 publications were retrieved after duplicates were removed. In total, there were 48,997 authors, 111 countries, 2372 journals, and 10,745 institutions.

### 3.2 Publications across all four decades

**Fig. 1:**
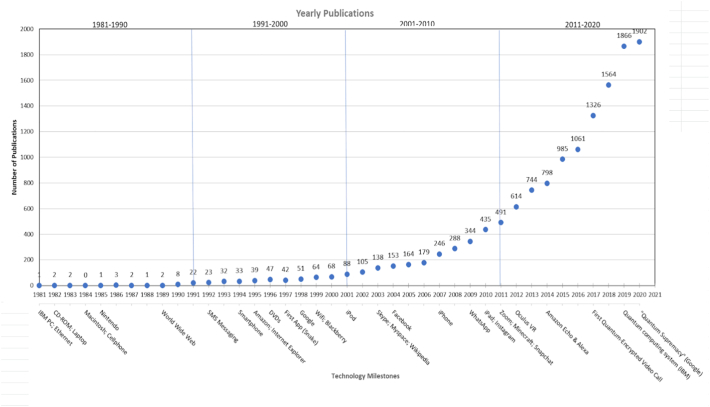
Yearly publications on psychotherapy and technology over the last 40 years based upon the 52 most frequent search terms. Note. 2020 represents 13.65% of publications across 40 years, and this percentage is based on data accumulated until October 20, 2020.

The evolution of the overall amount of publications on psychotherapy and technology over the last 40 years showed that there has been an increase in yearly publications from just one publication in 1981 to 1902 publications up to October 2020 (see Fig. 1). With regard to the growth of publications within each decade, the '80s were excluded due to a low number of publications. The growth was 209.09% from 1991 to 2000, 394.32% from 2001 to 2010, and 287.37% from 2011 to October 2020. Publications across decades yielded growth of 762.50% from the 1980s to the 1990s, 539.71% from the 90s to the 2000s, and 337.24% from the 2000s to the 2010s. Although the trend demonstrates a declining growth rate percentage across each decade, the number of publications did increase and were still tripling in size.

### 3.3 Authors, countries, journals, and institutions

The top author overall for the past four decades was Andersson G., followed by Cuijpers P., Carlbring P., Riper H., and Titov N. The top fifteen authors accounted for 1.9% of the total publications. The top country by publications overall was the USA, followed by the UK, Australia, Germany, and the Netherlands. The top fifteen countries accounted for 83.1% of the total publications. The top journal by publication overall was the *Journal of Medical Internet Research*, which was one of the first relevant journals, founded in 1990, followed by *Plos One*, *BMC Psychiatry*, *JMIR Mental Health*, and *Trials*. The top fifteen journals accounted for 18.6% of the total publications. Other journals that specifically focus on technology and psychology are Internet Interventions (ranked eleventh), Cyberpsychology Behavior and Social Networking (ranked 15th), and Telemedicine and *E*-Health (ranked 16th). The top institution overall was the Karolinska Institute, followed by the University of Washington, Linkoping University, Vrije University Amsterdam, and the University of Melbourne. The top fifteen institutions accounted for 8.8% of the total publications (see Table 1).

**Table 1:** Top fifteen authors, countries, journals, and institutions across four decades.

	Authors	N	%	LCS	Countries	N	%	LCS^a^	Journals	N	%	LCS	Institutions	N	%	LCS
1	Andersson G	276	0.3	5027	USA	5655	30.4	18,527	Journal of Medical Internet Research	561	4.0	365	Karolinska Inst	433	1.1	3882
2	Cuijpers P	148	0.2	2537	UK	1653	8.9	7165	Plos One	217	1.6	0	Univ Washington	290	0.8	1298
3	Carlbring P	137	0.2	2696	Australia	1475	7.9	6931	BMC Psychiatry	192	1.4	0	Linkoping Univ	289	0.7	3611
4	Riper H	109	0.1	1970	Germany	1113	6.0	3950	JMIR Mental Health	180	1.3	0	Vrije Univ Amsterdam	284	0.7	2066
5	Titov N	106	0.1	1729	Netherlands	892	6.0	4021	Trials	172	1.2	0	Univ Melbourne	267	0.7	888
6	Christensen H	104	0.1	1557	Canada	890	4.8	2072	BMJ Open	161	1.2	0	Kings Coll London	262	0.7	1376
7	Ebert DD	93	0.1	603	Sweden	673	3.6	4835	Journal of Affective Disorders	156	1.1	1265	Harvard Med Sch	217	0.5	395
8	Andrews G	87	0.1	1277	Peoples R China	572	3.1	586	JMIR MHealth and UHealth	140	1	0	Univ Calif San Francisco	213	0.5	712
9	Berger T	85	0.1	933	Spain	531	2.9	1364	Computers in Human Behavior	139	1	776	Univ Sydney	198	0.5	622
10	Botella C	80	0.1	666	Italy	510	2.7	1251	Behavior Research and Therapy	135	1	2355	Stanford Univ	195	0.5	622
11	Ljottson B	79	0.1	1091	Switzerland	351	1.9	1414	Internet Interventions	129	0.9	283	Univ Penn	194	0.5	667
12	Dear BF	71	0.1	766	France	322	1.7	768	Frontiers in Psychiatry	108	0.8	0	Univ Toronto	186	0.5	370
13	Riva G	63	0.1	264	South Korea	318	1.7	533	Frontiers in Psychology	107	0.8	0	Northwestern Univ	174	0.4	744
14	Mohr DC	61	0.1	586	Japan	275	1.5	447	Journal of Autism and Developmental Disorders	96	0.7	771	Univ Pittsburgh	168	0.4	564
15	Lindefors N	60	0.1	955	Denmark	211	1.1	623	Cyberpsychology Behavior and Social Networking	94	0.7	376	UCL	167	0.4	528
	Subtotal For Top Fifteen	1559	1.9	22,657		15,441	83.1	54,487		2587	18.6	6191		3537	8.8	18,345
	Total for all	79,956	100	265,283		18,580	100	66,437		13,940	100	48,761		39,907	100	129,808

a Note: LCS (Local Citation Scores) include citation frequencies within the collection of studies exclusively included in the bibliometric analysis.

### 3.4 Technology terms

Regarding the technology search terms, “internet*” had the largest number of publications across all four decades and was followed by “computer*,” “technolog*,” “online,” and “web*” (see Table 2). The top fifteen search terms accounted for 88.2% of publications over the last four decades.

**Table 2:** Total number of articles published by search term across over the last 40 years.

	Publications from 1981 to 2020	N	%
1	Internet*	2263	14.9
2	Computer*	1624	10.7
3	Technolog*	1439	9.5
4	Online	1325	8.7
5	Web*	1024	6.8
6	Video	800	5.3
7	Virtual Reality OR VR	800	5.3
8	App OR Apps	796	5.3
9	Phone* OR Telephone*	758	5.0
10	Cell* Phone* OR Smartphone* OR Smart Phone* OR Smart-phone* OR Mobile Phone* OR Mobile-phone* OR iPhone*	595	3.9
11	Digital	490	3.2
12	Machine Learning	440	2.9
13	Electronic*	383	2.5
14	Robo*	358	2.4
15	TeleHealth OR Tele-health OR Telehealth	255	1.7

Note. Percentages are based upon 13,934 publications with duplicates removed; however, as more than one search term can appear in the title and topic of an article, final calculations for Table 2 were based on a total of 15,141 publications.

Next, we analyzed the top fifteen technology terms per decade to search for trends and patterns over time (see Table 3). From 1981 to 1990, “computer*,” “web*,” “technolog*,” “video,” and “phone* OR telephone*” were the only published search terms. From 1991 to 2000, the top search term was “computer*,” followed by “phone* OR telephone*,” “technolog*,” and “video.” From 2001 to 2010, the top search term was “computer*,” followed by “internet*,” “phone* OR telephone*,” and “technolog*.” From 2011 to October 2020, the top search term was “internet*,” followed by “online*,” “technolog*,” and “computer*.” Overall, “computer*,” “web*,” “technolog*,” “video,” and “phone* OR telephone*” were consistently in the top fifteen most-published search term rankings from 1981 to 2020. “Virtual reality OR VR” and “app OR apps” were in the top fifteen for the last three decades.

**Table 3:** The top fifteen most published technology terms throughout the periods of 1981-1990, 1991-2000, 2001-2010, and 2011-2020.

	1981-1990	N	%	1991-2000	N	%	2001-2010	N	%	2011-2020	N	%
1	Computer*	14	58	Computer*	178	40.0	Computer*	397	17.6	Internet*	1883	15.2
2	Web*	5	21	Phone* OR Telephone*	52	11.7	Internet*	370	16.4	Online*	1219	9.8
3	Technolog*/ Video	2	8	Technolog*	40	9.0	Phone* OR Telephone*	223	9.9	Technolog*	1174	9.5
4	Phone* OR Telephone*	1	4	Video	37	8.3	Technolog*	220	9.7	Computer*	1033	8.3
5				App OR Apps	23	5.2	Video	158	7.0	Web*	858	6.9
6				Virtual Reality OR VR/ Digital	17	3.8	Web*	149	6.6	Virtual Reality OR VR	651	5.2
7				Web*	12	2.7	Virtual Reality OR VR	132	5.8	App OR Apps	649	5.2
8				Net	10	2.2	App OR Apps	124	5.5	Video	603	4.9
9				Virtual Environment	8	1.8	Online*	101	4.5	Cell Phone	573	4.6
10				Internet/Videophon*	7	1.6	Electronic*	73	3.2	Phone* OR Telephone*	482	3.9
11				Electronic*/Multimedia	6	1.3	Net	36	1.6	Digital	449	3.6
12				Machine Learning/ Microcomputer	4	0.9	Digital/Telepsych* OR Tele psych* OR Tele-psych*	24	1.1	Machine Learning	430	3.5
13				Online*/Cell Phone/Videoconference	3	0.7	Robo*/Multimedia/ Videoconference	22	1.0	Robo*	336	2.7
14				Interactive Video/ Computer System	2	0.4	Cell Phone	19	0.8	Electronic*	304	2.5
15				Artificial Intelligence/Telepsyche/Handheld/reSet/CD-ROM	1	0.2	Tele health OR Tele-health OR Telehealth"	18	0.8	Tele health OR Tele-health OR Telehealth"	236	1.9
	Total	24		446				2257			12,408	

Some search terms rose in ranking. “Internet*” appeared as a top fifteen search term in 2001-2010 as #2 and became the #1 search term in 2011-2020. “Online” appeared in 2001-2010 as #9 and rose to #2 in 2011-2020. Some search terms declined in ranking. “Phone* OR telephone*” declined over the last three decades from #2 in 1991-2000 to #10 in 2011-2020. “Video” declined form #4 in 1981-1990 to #8 in 2011-2020. Some search terms were only seen in the top fifteen for one decade. “Digital” and “virtual environment” were only in the top fifteen from 1991 to 2000; “net” was only in the top fifteen from 1991 to 2000, “electronic” was only in the top fifteen from 2001 to 2010, and “cell phone” was only in the top fifteen for 2011-2020.

**Fig. 2:**
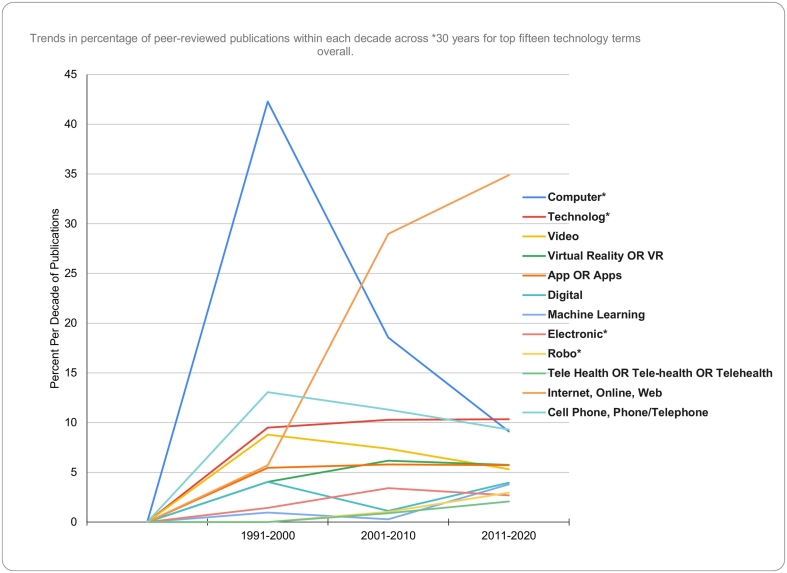
Trends in the percentage of peer-reviewed publications within each decade across the last three decades* for the top fifteen technology terms overall. *Note. The terms “internet,” “online,” & “web” were combined in one line and “cell phone” & “phone/telephone” in another line.

Using the top fifteen most published technology terms over the last 40 years, Fig. 2 plots the trends in percentages of peer-reviewed publications within each decade across the last three decades. The period from 1981 to 1990 was excluded from this analysis due to a low number of publications. In order to analyze unique patterns amongst the technology terms the following terms were combined: “internet,” “online,” and “web” and additionally “cell phone” and “phone OR telephone”. The search term “computer” demonstrated a declining trend representing 42.28% of total publications between 1991 and 2000, 18.55% in 2001-2010, and 9.1% for the most recent decade. The combined terms, “internet,” “online,” and “web” increased from 5.7% of publications to first position, representing 34.89% in 2011-2020. A group of technology terms had a steadier trend but a smaller number of publications (e.g.: the combined terms “cell phone” and “phone/telephone”, “technology”, “video”, “virtual reality or VR,” “apps,” “digital,” “machine learning,” “electronic,” “robo,” and “telehealth”).

## 4 Discussion

### 4.1 Growth

Understanding psychotherapy and technology publication trends across time can provide insight into the evolution and state of the science. While the growth of publications in technology and psychotherapy may seem obvious to those in the field, having a quantifiable idea of this growth may help anticipate future directions (Soares et al., 2020), help make terminology more consistent, and inform related policies that address the role of technology in mental health. The overall number of publications integrating psychotherapy and the 52 most common technology terms over the last 40 years showed an increase in publications from just one publication in 1981 to 1902 publications during the year 2020. The growth of publications within each decade and across decades has consistently tripled in size and demonstrated an upward trend that will continue to rise in the next few years, predictably at a higher rate after the COVID-19 pandemic.

Several factors have contributed to this growth. First, the rise of mobile ICTs and internet ubiquity has made technology more available and accessible, decreasing temporal and geographical barriers (Andersson et al., 2019; Mohr et al., 2017; United Nations, 2020a). Second, ongoing advances in technology have created more communication opportunities, allowing the massive adoption of social networking platforms which has contributed to an increased ability to provide psychotherapy through these means. Providing psychotherapy via social networking platforms has exhibited positive downstream effects, such as reduced costs and decreased study periods typically associated with research and delivery of psychotherapy utilizing technology (Andersson et al., 2019; Yan and Chen, 2015). Additionally, researchers around the world have gained interest in this field (Ritterband et al., 2006) which now receives support from both private and government funding agencies for new investigations and intervention dissemination (National Institute of Mental Health, 2018; Ritterband et al., 2006).

The growth in publications may also reflect the shifting paradigm within the field of psychotherapy. Researchers, practitioners, and clients are more open to technological innovations. A survey conducted during the COVID-19 pandemic reported that 64.2% of patients reported that they would be likely to continue utilizing remote treatment services in the future after the pandemic resolves (Guinart et al., 2020), Given the impact of social events on the evolution of scientific fields, it is expected the COVID-19 pandemic will further accelerate this growth as new and emerging technologies are adopted/incorporated (Asmundson and Taylor, 2020; Clipper, 2020; Wind et al., 2020).

### 4.2 Publication patterns

#### 4.2.1 Authors, countries, institutions

The authors with most publications in the field of psychotherapy and technology, considering all author positions in the dataset, over the past four decades were Andersson G., Cuijpers P., Carlbring P., Riper H., and Titov, N. The countries with the most publications were the USA, the UK, Australia, Germany, and the Netherlands. Consistent with trends by country, the top institutions were the Karolinska Institute, University of Washington, Linkoping University, Vrije University Amsterdam, and Melbourne University (which are situated within the first seven countries with the most publications). Interestingly, the top fifteen countries with more publications accounted for 83.1% of the total publications, reflecting a common trend in which a small minority of countries contribute the majority of global scientific literature (Fontelo and Liu, 2018).

The fact that few countries produce the majority of global scientific literature in this domain has several possible explanations. The first is a divergence in technology preferences and priorities amongst countries. Second, are international variances in economic resources and corresponding digital inequities. Third, are trends in international research collaborations. Support for technological research varies with national priorities (e.g., cultural affinity toward technology or tech-connectedness, perceived utility and ease of use, and facilitating infrastructure in place) (Connolly et al., 2020; Mahmood et al., 2009). The high cost of research is a primary barrier developing countries face. High-income countries have more economic resources and support from funding agencies to invest in science and consequently produce more scientific publications. Although the majority of the global population lives in developing countries, and even though internet access is expanding globally, as of 2020, only 19% of people in developing countries have internet access, compared to 87% in high-income countries (United Nations, 2020b). Advances in accessible ICTs offer methods of sharing scientific knowledge, supporting educational advances and research, while stimulating economic growth and social development (Miah and Omar, 2012); whereas unequal access across and within countries corresponds to social and economic inequalities (Ono and Zavodny, 2007). Because poverty within developing countries impacts access to ongoing technological advances, digital inequalities exacerbate the educational divide by wealth. Although international collaborative research has the potential to include developing countries, most collaborative growth occurs between countries already leading the literature output (Fontelo and Liu, 2018).

#### 4.2.2 Journals

The journals with the most publications were: *Journal of Medical Internet Research* (*JMIR*), *Plos One*, *BMC Psychiatry*, *JMIR Mental Health* (*JMIR-MH*), and *Trials*. Some of these journals are specifically oriented toward technology (e.g., *JMIR*) whereas others are more general (e.g., *BMC Psychiatry*). Also, there is a large discrepancy of time amongst the inception of many of these journals, including the technological journals. While *JMIR* was created 22 years ago, many other influential journals appeared recently (e.g., *Internet Interventions* began publishing 7 years ago). Of the top 15, only a few journals are distinctly related to technology, while the majority are not. Some of the journals with the most publications were disorder-specific (i.e., *Journal of Affective Disorders*; *Journal of Autism and Developmental Disorders*), which highlights the use of and high acceptability of technology in the treatment of these disorders.

### 4.3 Top trends

Regarding the technology search terms, “internet*” had the largest number of publications across all four decades, followed by “computer*,” “technolog*,” “online” and “web*.” The top 15 search terms accounted for 88.2% of publications over the last four decades. These terms include umbrella terms that reflect broad categories (e.g., “internet” may be an umbrella term for “online” and “web”), and some terms that represent unique technologies (e.g., “virtual reality,” “apps”). Although these umbrella terms provide a general insight into how technology is being harnessed in psychotherapy, it may misconstrue scientific research. For example, with the rise in demand for remote psychotherapy due to the COVID-19 pandemic, it may be challenging for researchers and clinicians to find “best practices” and guidelines for telemental health services when there are many interchangeable terms that refer to this modality (e.g., “videoconferencing”, “telehealth”, “telemental health”, “web-based”, “remote psychotherapy”, “digital mental health”, “mhealth”, “ehealth”, and “e-mental health”). The use of overlapping or interchangeable terms may contribute to a lack of clarity on what is being done, and this bibliometric analysis may contribute to determining the most frequent terms used to name specific technologies to avoid confusion in the field.

### 4.4 Terminology patterns

The frequency of the technology terms used in psychotherapy publications per decade was analyzed to search for patterns over time (see Table 3). Due to only having 22 publications from 1981 to 1990, this decade was excluded from pattern analysis. From 1991 to 2000, the top search term was “computer*,” followed by “phone* OR telephone*,” “technolog*,” and “video;” whereas “internet” was lower in the list. The top result of “computer*” compared to “internet” computers were the most prominent technology at the time, whereas the World Wide Web started in the mid-'90s and increased its reach in the 2000s (Internet Growth Statistics, 2020). Correspondingly, in the next decade (2001−2010), the top search term was also “computer*,” and “internet*” rose to second place. During 2011-2020, the top search term was “internet” which reflects the integral role the internet has assumed in the provision of psychotherapy services. Overall, “computer*,” “web*,” “technolog*,” “video,” and “phone* OR telephone*” were consistently in the top 15 most-published search term rankings from 1981 to 2020. While “internet” and “online” have grown in use over time, other terms such as “phone* OR telephone*” and “video” declined in ranking. “Cell phone” appeared only in the last decade, which is consistent with the massive adoption and increased capacity of this technology, as well as the wide proliferation of mental health applications (and rise in the term “apps”). The rapid expansion of mobile phone technology may be a possible explanation for the decline observed in the terms “phone* OR telephone*,” just as the decline in “video” may be associated with the emergence of “telehealth.”

### 4.5 Trends in percentage per decade

The trends in percentages of peer-reviewed publications within each decade across the last 30 years showed three notable patterns illustrated in Fig. 2. First, there is a declining trend for the term “computer.” Second, there is an upward trend for the combined terms, “internet,” “online,” and “web.” Third, we observe a steady but smaller proportion of publications for other terms (e.g.: the combined terms “cell phone” and “phone/telephone”, “technology”, “video”, “virtual reality or VR,” “apps,” “digital,” “machine learning,” “electronic,” “robo,” and “telehealth.”).

These trends in terminology may be due to the ever-changing nature of technology. “Internet,” “online,” and “web,” represent persistent, foundational technology that can function using fleeting technologies. While computers are integrally embedded within other electronics (e.g., watches), the use of computers has shifted toward other devices (e.g., smartphones, tablets). These findings can contribute to clarifying semantic inconsistencies previously identified by experts in the field (Smoktunowicz et al., 2020); it may be recommended to move away from using the term “computer” or “computerized” treatments given the decline in the use of the term and computers and to focus on terms that do not depend or highlight the device being used such as “internet” or “digital.”

### 4.6 Limitations and future directions

There are several limitations to the bibliometric analysis conducted. First, this study was limited to the WOS database's leading journal indices (the SCIE and SSCI) and English language. Although WOS is a large database, and the non-aggregated SCIE and SSCI indices optimize recall, precision, and reproducibility (Gusenbauer and Haddaway, 2020), in the future, researchers could expand the search scope to include other databases and languages. While many journals publish English translations of titles and abstracts of articles published in other languages, this may have inadvertently excluded publications conducted in other languages. For example, the present data may not comprehensively demonstrate China's growing contributions to the scientific literature (Fontelo and Liu, 2018).

A second possible limitation is that the search concluded in October of 2020. Although it is possible to estimate the likely number of publications for the remainder of the year, and anticipate growth based upon the past rates, 2020 publications may reflect a greater than expected increase in publications due to COVID-19 pandemic-related demands for providing and accessing psychotherapy services remotely.

Third, in an effort to represent trends in the field, analyses were based upon 52 technology terms (i.e., those terms with at least ten associated publications when combined with psychotherapy and mental health disorder keywords). Focus on this selection of terms may have overlooked technologies with fewer publications; however, the technologies with smaller numbers of publications would not have impacted the dominant trends.

Fourth, another limitation to consider is that the searches were limited to the field of psychotherapy, but more recently, psychotherapy has become increasingly integrated with the medical field, and by excluding the medical field other technology trends may have been overlooked (e.g., technologies used in integrated behavioral health care). Furthermore, we did not differentiate within the field of psychotherapy to note which were strictly psychotherapeutic interventions and which were self-help or preventative methods. Future research could explore variations in terminology usage across health fields, such as prevention science, psychiatry, and branches of the medical field.

Fifth, impact factor was not considered in the present study. This was done because the aim was to assess the number of publications that authors, journals, countries, and institutions had specifically in relation to articles that integrate psychotherapy and technology, whereas the impact factor relates to the overall publications of an author or journal.

Sixth, the searches and analysis were not filtered by type of population; therefore, it is unclear whether the articles included are related to psychotherapy with children, adolescents, parents, adults, or individuals versus groups. Future studies may want to explore the specific trends by type of population.

In addition, the aim of this bibliometric analysis was to examine the overall trends at the intersection of the fields of technology and psychotherapy, an investigation which necessitated examination of both technology-facilitated communication mediums and technology-delivered programming concurrently. This general approach permitted researchers to capture the broad picture of terminology used in the technology domain to date. As these technology formats vary operationally and conceptually, future studies addressing the demand for a common nomenclature may benefit from further refinement and differentiation between technology-related modalities. For example, differentiating between what is an actual videoconference in which a live therapist conducts therapy through a technology-mediated platform (e.g., Zoom) versus an intervention delivered through technology without a live therapist has proven to be difficult due to the “terminology chaos” (Barak, 2013).

## 5 Conclusions

The present study contributes to the existing literature on the science of psychotherapy and technology with an overall picture of increasing growth. The field emerged approximately 40 years ago and has rapidly evolved in the years since, accumulating approximately 14,000 peer-reviewed publications. Based upon the average growth of publication rates, it is anticipated that publications on psychotherapy and technology will continue to triple in size over the next decade. However, increased demand and interest due to the COVID-19 pandemic may accelerate this growth resulting in a paradigm shift in the field of psychology, moving from mostly face-to-face treatments to a majority of interventions assisted by or conducted exclusively through digital means.

The following are the supplementary data related to this article.

Appendix A Complete list of meta-analysis. Appendix A

Appendix B Complete list of Boolean search terms. Appendix B

Appendix C Expanded contents of Table 1. Appendix C

## Funding

This research did not receive any specific grant from funding agencies in the public, commercial, or not-for-profit sectors.

## Declaration of competing interest

The authors declare that they have no known competing financial interests or personal relationships that could have appeared to influence the work reported in this paper.
