# Supplementary material for: Technology and psychotherapeutic interventions: Bibliometric analysis of the past four decades
Source: Internet Interv. 2021 Jul 9;25:100425. doi: 10.1016/j.invent.2021.100425 (PMC8350597; doi:10.1016/j.invent.2021.100425)
Supplement: Appendix A — Complete list of meta-analysis. [file mmc1.docx]

Appendix A

*List of Systematic Reviews and Meta-Analyses with Corresponding Search Terms Used to Create the Initial Pool of Search Terms*

| References | Search Terms |
| --- | --- |
| Abd-Alrazaq, A. A., Alajlani, M., Alalwan, A. A., Bewick, B. M., Gardner, P., & Househ, M. (2019). An overview of the features of chatbots in mental health: A scoping review. International Journal of Medical Informatics, 103978. | “conversational agent” OR “chatbot” OR “chatterbot” OR “virtual agent” |
| Andersson, G., Cuijpers, P., Carlbring, P., Riper, H., & Hedman, E. (2014). Guided Internet‐based vs. face‐to‐face cognitive behavior therapy for psychiatric and somatic disorders: a systematic review and meta‐analysis. World Psychiatry, 13(3), 288-295. | “depression”, “panic disorder”, “social phobia”, “social anxiety disorder”, “generalized anxiety disorder”, “obsessive‐compulsive disorder”, “post‐traumatic stress disorder”, “specific phobia”, “hypochondriasis”, “bulimia”, “tinnitus”, “erectile dysfunction”, “chronic pain”, or “fatigue” AND “Internet” or “computer”, or “computerized” AND “randomized controlled trial” |
| Backhaus, A., Agha, Z., Maglione, M. L., Repp, A., Ross, B., Zuest, D., ... & Thorp, S. R. (2012). Videoconferencing psychotherapy: A systematic review. Psychological services, 9(2), 111. | “videoconferencing psychotherapy (VCP)” OR “Videoconferencing technology” OR “Telehealth” OR “Advanced technology” OR “computers” OR “smart phones” OR “virtual reality OR (“telemental health”)” OR “psychotherapy” and “telemedicine.” OR “mental health” OR “therapy” and “psychotherapy” OR “video and telehealth,” OR “video and telemedicine,” OR “teleconferencing” OR “Behavioral telehealth” OR “Computer-based treatment” OR “Interactive video” OR “interactive television” OR “Internet-based videoconferencing” OR “Remote counseling” OR “remote consultation” OR “remote treatment” OR “remote communication technologies” OR “remote methods” OR “Telecommunications” OR “telecommunications media” OR “Telehealth” OR “telehealth technology” OR “telehealth-mediated delivery” OR “Telemedicine” OR “telemedicine methods” OR “Telemental health” OR “telemental health services” OR “telemental healthcare” OR “Telepsychiatry” OR “Telepsychology” OR “rural telepsychology” OR “Telepsychotherapy” OR “Videoconference” OR “videoconference access” OR “videoconference treatment” OR “videoconferencing” OR “videoconferencing utility” OR “video-conferencing technology” OR “Videophones” OR “psychotherapy” OR “telemedicine.” OR “mental health,” “therapy,” and “psychotherapy” with the terms “video and telehealth,” “video and telemedicine,” and “teleconferencing” |
| Bakker, D., Kazantzis, N., Rickwood, D., & Rickard, N. (2016). Mental health smartphone apps: review and evidence-based recommendations for future developments. JMIR mental health, 3(1), e7. | “smart phone” OR “App” OR “web-app” OR “mobile” OR “SMS” OR "chat bot" OR "text*" OR "MHapp" OR "AnxietyCoach" OR "Behavioral Experiments" OR "Breathe" OR "DBT Diary Card and Skills Coach" OR " Depressive Prevention" OR "Happify" OR "HealthyHabits" OR "HealthyMinds" OR "HIAF" OR "iCouch CBT" OR "iCounselor" OR "iMoodJournal" OR "In Hand" OR "Mind Shift" OR "MoodKit" OR "Moodlytics" OR "Moody Me" OR "Pacifica" OR "Pocket CBT" OR "SAM" "Smiling Mind" OR "Stress & Anxiety Companion" OR "SuperBetter" OR "ThinkHappy" OR "What's Up?" OR "WorkOut" OR "WorryTime" OR "Kokoro" OR "SmartCAT" OR "Personalized Real-Time Intervention for Stabilizing Mood (PRISM)" OR "focus" OR "Actissist" OR "Alcohol Comprehensive Health-Enhancement Support System" OR "STUB IT" OR "Happy Ending" OR "Depression Test" OR "Depression Tracker and Diary" OR "Emotion" OR "Life Robot- Fight Depression" OR "Mood tracker- depression" OR "Start" OR "iDepression Tracker" OR "Depression Inventory" OR "Depression Journal" OR “Augmentative and Alternative Communication (AAC) Device” |
| Bendig, E., Erb, B., Schulze-Thuesing, L., & Baumeister, H. (2019). The next generation: chatbots in clinical psychology and psychotherapy to foster mental health–a scoping review. *Verhaltenstherapie*, 1-13. | “chatterbot” OR “chatbot” OR “social bot” OR “conversational agent” OR “softbot” OR “virtual agent” OR “software agent” OR “conversational agent” OR “automated agent” AND “psych*” OR “counseling” OR “mental health” OR “psychotherapy” OR “therap*” OR “mental*” OR “clinical psychology” |
| Carbonell, X., Guardiola, E., Beranuy, M., & Bellés, A. (2009). A bibliometric analysis of the scientific literature on Internet, video games, and cell phone addiction. *Journal of the Medical Library Association: JMLA*, *97*(2), 102. | ‘‘Internet” OR ‘‘Cellular Phone’’ OR ‘‘Video Games’’ OR ‘‘Computer Systems” OR ‘‘Computers’’AND ‘‘Impulse Control Disorders’’ OR ‘‘Obsessive-Compulsive Disorder’’ OR ‘‘Anxiety Disorders” OR ‘‘Mood Disorders’’ OR ‘‘Impulsive Behavior’’ OR ‘‘Behavior Addictive” |
| Cuijpers, P., Van Straten, A., Andersson, G., & Van Oppen, P. (2008). Psychotherapy for depression in adults: a meta-analysis of comparative outcome studies. *Journal of consulting and clinical psychology*, *76*(6), 909. | “Psychotherapy” OR “psychological treatment” OR “cognitive therapy” OR “behavior therapy” OR “interpersonal therapy” OR “reminiscence” and “life review” |
| David, D., Matu, S. A., & David, O. A. (2014). Robot-based psychotherapy: Concepts development, state of the art, and new directions. International Journal of Cognitive Therapy, 7(2), 192-210. | “robot-based psychotherapy” OR “robotics-based psychotherapy” OR “Robo-Therapist” OR “Robo-Mediator” OR “Robo-Assistant” OR “Robotic psychology” OR “robopsychology” OR “assistive robots” OR “interactive robots” OR “social robots” OR “Robot-Based CBT” OR “CBT Virtual Agent” OR “Virtual Agent” OR “Help4Mood” OR “Psychological Pills Virtual Agent” OR “Apps PsyPills” OR “Robo-RETMAN” OR “Robo-Animals” OR “robotic dog AIBO” OR “NeCoRo” OR “Bandit OR Nabaztag” OR “Nao robot OR” |
| Depp, C. A., Mausbach, B., Granholm, E., Cardenas, V., Ben-Zeev, D., Patterson, T. L., ... & Jeste, D. V. (2010). Mobile interventions for severe mental illness: design and preliminary data from three approaches. The Journal of nervous and mental disease, 198(10), 715. | “Personalized Real-Time Intervention for Stabilizing Mood (PRISM)” OR “Mobile Assessment and Therapy for Schizophrenia (MATS)” OR “Skills Training and Empowerment Program (STEP)” OR “Automated momentary assessment” OR “Computer initiated text messaging” OR “Live telephone interaction” |
| Donker, T., Petrie, K., Proudfoot, J., Clarke, J., Birch, M. R., & Christensen, H. (2013). Smartphones for smarter delivery of mental health programs: a systematic review. Journal of medical Internet research, 15(11), e247. | “mobile applications” OR “mobile mental” OR “health” OR “mobile phones” OR “self-help” OR “depression” OR “anxiety” OR “stress” OR “substance use” OR “mental disorder” OR “mental ilness*” OR “depress*” OR “anxi*” OR “Stress*” OR “panic*” OR “agoraphob*” OR “mood” OR “phobi*” OR “social anxi*” OR “mobile health” OR “cell*” OR “phone*” OR “mobile phone*” OR “sms*” OR “text*” or “short* messag*” OR “mms*” OR “mobile app*” OR “smartphone*” OR “mobile device*” OR “ipad*” OR “iphone*” OR “ipod*” OR “pda” OR “personal digital assistance*” OR “tablet*” OR “handheld computer” OR “electronic diar*” OR “microcomputer” AND “preventi*” OR “ecological momentary assessment” OR “therap*” OR “program*” OR “psychotherap*” OR “treatment” OR “evaluation” OR “trial” OR “randomized control*” OR “protocol” OR “proof” OR “case*” OR “pilot” |
| Firth, J., & Torous, J. (2015). Smartphone apps for schizophrenia: A systematic review. JMIR mHealth and uHealth, 3(4), e102. | “ClinTouch” OR “FOCUS" OR “WellWave” OR “PeerFIT” OR “AIR" OR “smartphone*” or “mobile phone*” or “cell phone” or “iPhone” or “mobile app*” or “phone app*” |
| Guy, S., Ratzki-Leewing, A., & Gwadry-Sridhar, F. (2011). Moving beyond the stigma: Systematic review of video games and their potential to combat obesity. *International journal of hypertension*, *2011*. | “Obesity” OR “overweight” OR “physical activity” OR “fitness” OR “exercise” OR “energy” OR “expenditure” OR “heart rate” OR “energy metabolism” OR “nutrition” OR “BMI” OR “diet” OR “video gam∗” OR “exergam∗” OR “active video gam∗” OR “active computer gam∗” OR “new generation computer gam∗” OR “exertainment” OR “active gam∗” and “computer gam∗” |
| Hollis, C., Falconer, C. J., Martin, J. L., Whittington, C., Stockton, S., Glazebrook, C., & Davies, E. B. (2017). Annual Research Review: Digital health interventions for children and young people with mental health problems–a systematic and meta‐review. Journal of Child Psychology and Psychiatry, 58(4), 474-503. | “Mobile Applications” OR “Telemedicine” OR “Blogging” OR “Inventions” OR “CD-ROM” OR “Internet” OR “Computers” OR “Electronics” OR “Electronic Mail” OR “Text Messaging” OR “Web Browser” OR “Virtual Reality Exposure Therapy” OR “Video Games” OR “Cell Phones” OR “Computers” OR “Handheld” OR “Microcomputers” OR “Social Media” AND “Adolescent” OR “Child” OR “Preschool” OR “Minors” OR “Students” AND “Mental Health” OR “Mental Disorders” OR “Anxiety” OR “Anxiety Disorders” OR “Depression” OR “Dysthymic Disorder” OR “Depressive Disorder” OR “Major” OR “Mood Disorders” OR “Seasonal Affective Disorder” OR “Depressive Disorder” OR “Panic” OR “Panic Disorder” OR “Phobic Disorders” OR “Obsessive-compulsive disorder” OR “Stress Disorders” OR “Post-Traumatic” OR “Eating Disorders” OR “Feeding and Eating Disorders of Childhood” OR “Anorexia Nervosa” OR “Body Dysmorphic Disorders” OR “Bulimia Nervosa” OR “Binge-Eating Disorder” OR “Attention Deficit Disorder with Hyperactivity” OR “Conduct Disorder” OR “Alcoholism” OR “Alcohol addiction” OR “Substance-related disorders” OR “Autistic Disorder” OR “Asperger Syndrome” OR “Tourette Syndrome” OR “Psychotic Disorders” OR “Schizophrenia” OR “Bipolar Disorder” OR “Cyclothymic Disorder” OR “Self-Injurious Behavior” OR “Sleep Initiation and Maintenance Disorders” OR “Adjustment Disorders” AND “TI=Narrative review” OR “Scoping review” OR “systematic review” OR “meta-analysis” OR “meta-analytical review” OR “Mobile Applications” OR “Telemedicine” OR “Blogging” OR “Inventions” OR “CD-ROM” OR “Internet” OR “Computers” OR “Electronics” OR “Electronic Mail” OR “Text Messaging” OR “Web Browser” OR “Virtual Reality Exposure Therapy” OR “Video Games” OR “Cell Phones” OR “Computers” OR “Handheld” OR “Microcomputers” OR “Social Media” OR “attitude to computers” OR “audiovisual aid” OR “audiovisual equipment” OR “communication software” OR “computer assisted therapy” OR “computer program” OR “computer system” OR “computer” OR “decision support system” OR “e-mail” OR “human computer interaction” OR “information technology” OR “internet” OR “mobile phone” OR “multimedia” OR “exp optical disk” OR “personal digital assistant” OR “social media” OR “telecommunication” OR “teleconsultation” OR “exp telehealth” OR  “telemedicine” OR “telemonitoring” OR “telephone” OR “telepsychiatry” OR “teletherapy” OR “text messaging” OR “video disk” OR “videotape” OR “audiotapes” OR “audiovisual communications media” OR “communications media” OR “computer applications” OR “exp computer assisted instruction” OR “computer assisted therapy” OR “computer attitudes” OR “computer literacy” OR “computer mediated communication” OR “computer software” OR “computer training” OR “computers” OR “digital video” OR “educational audiovisual aids” OR “electronic communication” OR “exp human computer interaction” OR “hot line services” OR “human computer interaction” OR “hypermedia” OR “information technology” OR “instructional media” OR “internet” OR “exp mobile devices” OR “exp multimedia” OR “online therapy” OR “programmed instruction” OR “exp social media” OR “exp social networks” OR “telecommunications media” OR “telemedicine” OR “telemetry” OR “exp telephone systems” OR “videotapes” |
| Huguet, A., Rao, S., McGrath, P. J., Wozney, L., Wheaton, M., Conrod, J., & Rozario, S. (2016). A systematic review of cognitive behavioral therapy and behavioral activation apps for depression. PloS one, 11(5), e0154248. | "Depression Test & Tracker" OR "MoodTools- Depression Aid" OR "Overcome the Depression pro" OR "Anti-Depression" OR "Activity Diary" OR "Depression" OR "Depression CBT Self- Help Guide" OR "Depression Cure- The free 12 week course" OR "iCounselor: Depression" OR "Mood Master Anti- Depression App" OR "Mood Sentry" OR "Positive Activity" OR "Jackpot" OR "eCBT Mood" OR "Beat Depression Hypnosis Audio" OR "Depression Cure Hypnosis" OR "Depression Mood Booster" OR "Fight Depression" OR "From Depression to Hope" OR "MoodSpace" OR "Vital Tones Depression" OR "Yoga for Depression" OR "Yoga Helps Relieve Depression" OR "Life Robot- Fight Depression" OR “Depression Relief and Mood–HappyApp” OR “Mood Elevator & Support” OR “Beat Depression Hypnosis System” OR “Depression Help Brainwave” OR “Depression Inventory” OR “Heal Depression Hypnosis” OR “The Mindful Way Through Depression” OR “Black Rainbow: How to Beat Depression” OR “CESD Depression Test” OR “Depression Diagnosis Doctor” OR “Depression Eval Questionnaire” OR “Depression Screening Test” OR “Depression Test” OR “Depression Test” and “Treatment” OR “Depression Tracker & Diary” OR “Depression Test Pro” OR “Am I Depressed” OR “Do I have Depression” OR “Happy App” OR “Zung” OR “Are You at Risk for Depression?” OR “Depression Calculator” OR “Depression Screening” OR “Learn About Depression” OR “Major Depression Checker” OR “Sad Scale Lite” OR “STAT Depression Screening PHQ 9” OR “The Depression Predictor” OR “Depression Test & Tracker” OR “Conquering Depression OR Dealing with Depression” OR “Depression and How to Stop it” OR “Depression & Psychology” OR “Depression Definition” OR “Depression Healing” OR “Depression Information” OR “Depression Management” OR “Depression Symptoms” OR “Depression Symptoms and Signs OR Depression: An Overview” OR “Depression: Natural Remedies” OR “Fitness Against Depression” OR “Help with Depression” OR “How to get Over Depression” OR “Physical Symptoms Depression” OR “Reduce Depression” OR “The Key to Happiness” OR “Black Rainbow: How to Beat Depression” OR “Beat Depression” OR “Depression Treatment” OR “Overcoming Depression” OR “Depression 101” OR “Depression Advice” OR “NIH Depression Information” OR “Ten Tips to Ease Depression” OR “The Depression Predictor” OR “Are You at Risk for Depression?” OR “Depression Preview” OR “Dealing with Depression” OR “You Are Important” OR “A Guiding Light” OR “Acupuncture Against Depression” OR “Afternoon in Depression” OR “Best Depression Quotes” OR “dePRESSION” OR “Depression Quotes Wallpaper” OR “Depression- Acupuncture” OR “Depressive and Sad Wallpaper” OR “Endless Depression” OR “Get Rid of Depression with Chinese Massage Points” OR “Guide to Depression SelfHelp” OR “How to Beat Depression” OR “Sad Quotes Wallpaper” OR “Sadness and Depression Quotes” OR “Secret of Happiness” OR “Self-Help for Depression” OR “MoodSpace” OR” Life Robot- Fight Depression” OR “Depression Management” OR “DepressPill Game for Happy” OR “Joker” OR “Anti-Depression Grocery List” OR “Depression Fighter- A Practical Christian Guide” OR “Surviving Depression” |
| Jackson, D., Roberts, G., Wu, M. L., Ford, R., & Doyle, C. (2016). A systematic review of the effect of telephone, internet or combined support for carers of people living with Alzheimer’s, vascular or mixed dementia in the community. Archives of gerontology and geriatrics, 66, 218-236. | “information communication technology (ICT)” OR “dementia*” OR “Alzheimer*” OR “cognit*” AND “caregiver*” AND “telephone*” OR “computer*” OR “internet*” OR “telehealth” OR “telephone” OR “ehealth” OR “electronic” OR “computer” AND “dementia” AND “carer. (dementia AND telephone)” AND “carer” OR “care giver” OR “tele” OR “internet” OR “computer” OR “network*” |
| Moore, D. J., Pasipanodya, E. C., Umlauf, A., Rooney, A. S., Gouaux, B., Depp, C. A., ... & Montoya, J. L. (2018). Individualized texting for adherence building (iTAB) for methamphetamine users living with HIV: A pilot randomized clinical trial. Drug and alcohol dependence, 189, 154-160. | “individualized texting for adherence building (iTAB)” |
| Opriş, D., Pintea, S., García‐Palacios, A., Botella, C., Szamosközi, Ş., & David, D. (2012). Virtual reality exposure therapy in anxiety disorders: A quantitative meta‐analysis. Depression and anxiety, 29(2), 85-93. | “Virtual reality exposure therapy (VRET)” OR “VRET’’ OR “virtual reality and anxiety’’ OR “virtual reality and exposure’’ OR “virtual reality and phobia’’ OR “virtual reality and panic disorder’’ OR ‘‘virtual reality and generalized anxiety disorder’’ OR ‘‘virtual reality and obsessive compulsive disorder’’ OR ‘‘virtual reality and posttraumatic stress disorder" OR “Computer Technology based treatments” OR “computer-aided psychotherapy” OR “Internet-based treatments” OR “behavioral therapy augmented by virtual reality exposure” OR “cognitive-behavioral therapy augmented by virtual reality exposure” |
| Primack, B. A., Carroll, M. V., McNamara, M., Klem, M. L., King, B., Rich, M., ... & Nayak, S. (2012). Role of video games in improving health-related outcomes: a systematic review. American journal of preventive medicine, 42(6), 630-638. | “'video game” OR “video games” OR “video gamer” OR “video gamers” OR “video gaming” OR “videogame” OR “videogames” OR “videogamer” OR “videogamers” OR “videogaming” OR “computer game” OR “computer games” OR “computer gamer” OR “computer gamers” OR “computer gaming” OR “online game” OR “online games” OR “online gamer” OR “online gamers” OR “online gaming” OR “game system” OR “games system” OR “gamer system” OR “gamers system” OR “gaming system” OR “game systems” OR “games systems” OR “gamer systems” OR “gamers systems” OR “gaming systems” OR “arcade game” OR “arcade games” OR “arcade gamer” OR “arcade gamers” OR “arcade gaming” OR “playstation” OR “playstations” OR “interactive game” OR “interactive games” OR “interactive gamer” OR “interactive gamers” OR “interactive gaming” OR “gamer” OR “gamers” OR “game console” OR “game consoles” OR “gaming console” OR “gaming consoles” OR “digital game” OR “digital games” OR “digital gamer” OR “digital gamers” OR “digital gaming” OR “handheld game” OR “handheld games” OR “handheld gamer” OR “handheld gamers” OR “handheld gaming” OR “console game” OR “console games” OR “console gamer” OR “console gamers” OR “console gaming” OR “multiplayer” OR “multiplayers” OR “gameplay” OR “gameplayer” OR “gameplayers” OR “gameplaying” OR “game boy” OR “game boys” OR “game cube” OR “game cubes” OR “nintendo” OR “xbox” OR “mmorpg” OR “atari” OR “space invader” OR “space invaders” OR “death race” OR “pac man” OR “battlezone” OR “astrocade” OR “donkey kong” OR “coleco” OR “tetris” OR “super mario” OR “sonic the hedgehog” OR “street fighter” OR “mortal kombat” OR “pokemon” OR “frogger” OR “dreamcast” OR “grand theft auto” AND “random*” OR “placebo*” OR “placebo'/de” OR “placebo effect” OR “double blind” OR “double blinding” OR “double blind” |
| Soares, E. E., Thrall, J. N., Stephens, T. N., Rodriguez Biglieri, R., Consoli, A. J., & Bunge, E. L. (2020). Publication trends in psychotherapy: Bibliometric analysis of the past 5 decades. American Journal of Psychotherapy, 73(3), 85-94. | “psychotherap*” OR “intervention*” OR “therap*” OR “treatment*” OR “program*” OR “manual*” OR “protocol*” |
| Torous, J., Levin, M. E., Ahern, D. K., & Oser, M. L. (2017). Cognitive behavioral mobile applications: clinical studies, marketplace overview, and research agenda. *Cognitive and Behavioral Practice*, *24*(2), 215-225. | “mobile app” OR “mobile” OR “smartphone” |
| Turner, W. A., & Casey, L. M. (2014). Outcomes associated with virtual reality in psychological interventions: where are we now?. Clinical psychology review, 34(8), 634-644. | “virtual reality” OR “VR” OR “technology” OR “eHealth” OR “review” OR “methodology” OR “meta-analysis” OR “head-mounted displays (HMD)” OR “VR glasses/goggles quality” OR “motion tracking technology” OR “Oculus Rift” OR “Sony HMZ-T2 Personal 3D Viewer” OR “virtual environment (VE)” OR “e game” OR “virtual” OR “reality” OR “VR” OR “Wii” OR “Xbox” OR “PlayStation” OR “behavioural VR based exposure” OR “VR skills training” OR “VR cognitive behavioural therapy” OR “VR occupational therapy/physical rehabilitation” OR “active technologically based interventions” |
| Villani, D., Carissoli, C., Triberti, S., Marchetti, A., Gilli, G., & Riva, G. (2018). Videogames for emotion regulation: A systematic review. Games for Health Journal, 7(2), 85-99. | “emot*” AND “regulat*” AND “video game” OR “videogame”; “emot*” AND “regulat*” AND “serious game”; “emot*” AND “manag*” AND “video game” or “videogame” OR “emot*” AND “manag*” AND “serious game”; “emot*” AND “cop*” AND “video game” or “videogame”; “emot*” AND “cop*” AND “serious game”; “positive AND “affect” AND “video game” or “videogame”; “positive” AND “affect” AND “serious game”; “positive” AND “emotion” AND “video game” or “videogame”; “positive” AND “emotion” AND “serious game” AND “mood” AND “video game” or “videogame”; “mood” AND “serious game” |
