# Supplementary material for: Technology and psychotherapeutic interventions: Bibliometric analysis of the past four decades
Source: Internet Interv. 2021 Jul 9;25:100425. doi: 10.1016/j.invent.2021.100425 (PMC8350597; doi:10.1016/j.invent.2021.100425)
Supplement: Appendix B — Complete list of Boolean search terms. [file mmc2.docx]

Appendix 2. List Of Technology Search Terms and # of Articles

|  | Technology Search Term | # of Articles | % |
| --- | --- | --- | --- |
| 1 | **“Internet*”** | 2263 | 14.94% |
| 2 | **“Computer*”** | 1624 | 10.72% |
| 3 | **“Technolog*” Not “Reproductive”** | 1439 | 9.50% |
| 4 | **“Online*”** | 1325 | 8.75% |
| 5 | **“Web*”** | 1024 | 6.76% |
| 6 | **“Video” Not "Videoconferenc*" Or "Video-conferenc*" Or "Video Conferenc*" Or "Video Gam*" Or "Videogam*" Or "Video-gam*"** | 800 | 5.28% |
| 7 | **"Virtual Reality" Or "Vr"** | 800 | 5.28% |
| 8 | **"App" Or "Apps" Not "Ps1" Or "Mouse" Or "Mice" Or "Amyloid Precursor Protein"** | 796 | 5.26% |
| 9 | **“Phone*” Or “Telephone*” Not "Cell* Chone*" Or "Smartphone*" Or "Smart Phone*" Or "Smart-phone*" Or "Mobile Phone*" Or "Mobile-phone*" Or "Iphon*" Or “Phonem*” Or "Phonetic*"** | 758 | 5.00% |
| 10 | **"Cell* Phone*" Or "Smartphone*" Or "Smart Phone*" Or "Smart-phone*" Or "Mobile Phone*" Or "Mobile-phone*" Or "Iphon*"** | 595 | 3.93% |
| 11 | **“Digital”** | 490 | 3.23% |
| 12 | **"Machine Learning"** | 440 | 2.90% |
| 13 | **"Electronic*" Not "Health Record*" Or "Medical Record*" Or "Electronic Record*" Or "Electronic Dance"** | 383 | 2.53% |
| 14 | **“Robo*” Not “Prostatectom*” Or “Laparoscopic” Or “Hysterectomy” Or “Zebrafish”** | 358 | 2.36% |
| 15 | **"Tele Health Or Tele-health Or Telehealth"** | 255 | 1.68% |
| 16 | **“Net”** | 202 | 1.33% |
| 17 | **"Video Gam*" Or "Videogam*" Or "Video-gam*"** | 134 | 0.88% |
| 18 | **"Text Messag*"** | 127 | 0.84% |
| 19 | **“Mhealth”** | 109 | 0.72% |
| 20 | **“Videoconferenc*”** | 107 | 0.71% |
| 21 | **“Tablet” Not “Oral” Or “Bioavailability” Or “Pharma*” Or “Medication*” Or “Chewable”** | 100 | 0.66% |
| 22 | **“Wearable* Not "Pressure Injur*" Or "Chronic Obstructive Pulmonary Disease" Or "Copd"** | 97 | 0.64% |
| 23 | **“Ehealth”** | 97 | 0.64% |
| 24 | **“Multimedia”** | 90 | 0.59% |
| 25 | **"Artificial Intelligence" Or "Ai"** | 86 | 0.57% |
| 26 | **"Telepsych*" Or "Tele Psych*" Or "Tele-psych*"** | 82 | 0.54% |
| 27 | **"Virtual Environment*"** | 74 | 0.49% |
| 28 | **"E-mental Health"** | 65 | 0.43% |
| 29 | **“Cyber”** | 54 | 0.36% |
| 30 | **"E-mail" Or "Email" Or "E Mail"** | 51 | 0.34% |
| 31 | **Exergam*** | 47 | 0.31% |
| 32 | **"Telemental Health*" Or "Tele-mental Health*" Or "Tele Mental Health*"** | 33 | 0.22% |
| 33 | **"Online Gam*"** | 23 | 0.15% |
| 34 | **"Assistive Robot*"** | 22 | 0.15% |
| 35 | **"E-therap*" Or "E Therap*"** | 21 | 0.14% |
| 36 | **"Computer Gam*"** | 19 | 0.13% |
| 37 | **"Handheld*" Or "Hand-held" Or "Hand Held"** | 19 | 0.13% |
| 38 | **“Cd-rom*”** | 17 | 0.11% |
| 39 | **"Conversational A*"** | 16 | 0.11% |
| 40 | **“Blog*”** | 15 | 0.10% |
| 41 | **“Gamer*”** | 14 | 0.09% |
| 42 | **"Wii"** | 12 | 0.08% |
| 43 | **"Teletherap*" Or "Tele Therap*" Or "Tele-therap*"** | 12 | 0.08% |
| 44 | **"Videophon*"** | 11 | 0.07% |
| 45 | **“Chatbot*”** | 10 | 0.07% |
| 46 | **"Interactive Video*"** | 8 | 0.05% |
| 47 | **“Reset”** | 6 | 0.04% |
| 48 | **"Computer System*"** | 5 | 0.03% |
| 49 | **“Microcomputer*”** | 4 | 0.03% |
| 50 | **"Massively Multiplayer Online Role-playing Game*" Or "Mmorpg"** | 3 | 0.02% |
| 51 | **"Advanced Technolog*"** | 3 | 0.02% |
| 52 | **“Gameplay*”** | 2 | 0.01% |
| 53 | "Mobile Phone*” | 0 | 0.0% |
| 54 | “Telephon*” | 0 | 0.0% |
| 55 | “Smartphon*” | 0 | 0.0% |
| 56 | “Videogam*” | 0 | 0.0% |
| 57 | "Active Gam*" | 0 | 0.0% |
| 58 | “Muse” | 0 | 0.0% |
| 59 | “Bits” | 0 | 0.0% |
| 60 | “Relational Agent*” | 0 | 0.0% |
| 61 | "Conference Call*" | 0 | 0.0% |
| 62 | "Behavioral Intervention Technolog*" | 0 | 0.0% |
| 63 | "Interactive Gam*" | 0 | 0.0% |
| 64 | “Smartwatch” | 0 | 0.0% |
| 65 | “Xbox” | 0 | 0.0% |
| 66 | “Help4mood” | 0 | 0.0% |
| 67 | "Gam* System*" Not "Gamma" Not "Gambling" | 0 | 0.0% |
| 68 | "Smart Glasses" | 0 | 0.0% |
| 69 | "Apple Watch" | 0 | 0.0% |
| 70 | "Head-mounted Displays" Or "Hmd" | 0 | 0.0% |
| 71 | “E-counseling” | 0 | 0.0% |
| 72 | "I-therapy" | 0 | 0.0% |
| 73 | "Interactive Robot*" | 0 | 0.0% |
| 74 | "Oculus Rift" | 0 | 0.0% |
| 75 | "Electronic Mail" | 0 | 0.0% |
| 76 | “I-health” | 0 | 0.0% |
| 77 | “Touchpoints” | 0 | 0.0% |
| 78 | "Video Teleconference" Or "Vtc" | 0 | 0.0% |
| 79 | "Motion Tracking Technolog*" | 0 | 0.0% |
| 80 | "Gam* Console*" | 0 | 0.0% |
| 81 | “Bandit” | 0 | 0.0% |
| 82 | "Interactive Television" | 0 | 0.0% |
| 83 | “I-attachment” | 0 | 0.0% |
| 84 | "Dialog* System" | 0 | 0.0% |
| 85 | "Google Glasses" | 0 | 0.0% |
| 86 | "Game Console*" | 0 | 0.0% |
| 87 | "E Game" | 0 | 0.0% |
| 88 | "Game Boy*" | 0 | 0.0% |
| 89 | “Playstation*” | 0 | 0.0% |
| 90 | "Software Agent" | 0 | 0.0% |
| 91 | "I-act" | 0 | 0.0% |
| 92 | "Console Gam*" | 0 | 0.0% |
| 93 | "Conversational System*" | 0 | 0.0% |
| 94 | "Dhi" | 0 | 0.0% |
| 95 | "Nao Robot*" | 0 | 0.0% |
| 96 | "Active Computer Gam*" | 0 | 0.0% |
| 97 | "Active Technologically Based Interventions" | 0 | 0.0% |
| 98 | "Arcade Gam*" | 0 | 0.0% |
| 99 | "Assistance Technolog*" | 0 | 0.0% |
| 100 | "Augmented By Virtual Reality Exposure" | 0 | 0.0% |
| 101 | "Automated Agent" | 0 | 0.0% |
| 102 | “Chatterbot” | 0 | 0.0% |
| 103 | "Dmhi" | 0 | 0.0% |
| 104 | “E-psychotherapy” | 0 | 0.0% |
| 105 | “Exertainment” | 0 | 0.0% |
| 106 | "Game Cube*" | 0 | 0.0% |
| 107 | "I-applied Relaxation" | 0 | 0.0% |
| 108 | “Mybivy’ | 0 | 0.0% |
| 109 | “Nabaztag” | 0 | 0.0% |
| 110 | “Necoro” | 0 | 0.0% |
| 111 | "New Generation Computer Gam∗" | 0 | 0.0% |
| 112 | "Psychological Pills Virtual Agent" | 0 | 0.0% |
| 113 | “Reset-o” | 0 | 0.0% |
| 114 | "Social Bot" | 0 | 0.0% |
| 115 | “Softbot” | 0 | 0.0% |
| 116 | “Thync” | 0 | 0.0% |
| 117 | "Voice Over Internet Protocol" Or "Voip" | 0 | 0.0% |

*For the Web of Science (WOS) search, every Technology search term was run with the same corresponding Psychotherapy and Mental Disorder search terms. For simplicity, the Psychotherapy and Mental Disorder search terms were only added to the first row.
